# Environmental Quality of Italian Marine Water by Means of Marine Strategy Framework Directive (MSFD) Descriptor 9

**DOI:** 10.1371/journal.pone.0108463

**Published:** 2014-09-24

**Authors:** Chiara Maggi, Serena Lomiri, Bianca Di Lorenzo, Marco d’Antona, Maria Teresa Berducci

**Affiliations:** ISPRA, Institute for Environmental Protection and Research, Roma, Italy; Università della Calabria, Italy

## Abstract

ISPRA, on behalf of the Italian Ministry of Environment, carried out the initial assessment of environmental quality status of the 3 Italian subregions (Mediterranean Sea Region) on Descriptor 9. The approach adopted to define the GES started to verify that contaminants in fish and other seafood for human consumption did not exceed levels established by Community legislation (Reg. 1881/2006 and further updates). As the Marine Strategy Framework Directive (MSFD) requires to use health tools to assess the environment, Italy decided to adopt a statistical range of acceptance of thresholds identified by national (D.Lgs. 152/2006 concerning water quality required for mussel farms) and international legislation (Reg. 1881/2006 and further updates), which allowed to use the health results and to employ them for the assessment of environmental quality. Italy proposed that Good Environmental Status (GES) is achieved when concentrations are lower than statistical range of acceptance, estimated on samples of fish and fishery products coming from only national waters. GIS-based approach a to perform different integration levels for station, cell’s grid and years, was used; the elaborations allowed to judge the environmental quality good.

## Introduction

Marine waters have traditionally been used by population for different activities (e.g. fishing, aquaculture, shipping, tourism, discharges from agriculture and urban areas). Currently, new activities are being developed or increasing (e.g. renewable energies, extraction of minerals, etc.), and thus competing for space with traditional uses, causing many spatial conflicts and increasing human impacts on marine ecosystems [1], [2], [3]. On the other hand, numerous anthropogenic sources such as land-based industrial activity, urban discharge, pesticide use, nuclear accidents and discharges, aquaculture, heavy shipping lines might be responsible for elevated levels of contaminants in sediment and biota, including species for human consumption.

To face this status the European Parliament and the Council of the European Union on 17 June 2008 have issued the Framework Directive 2008/56/EC (MSFD, [4]) on the Strategy for the Marine Environment, subsequently implemented in Italy with the Legislative Decree n. 190 of 13 October 2010 [5].

The aim of the Marine Strategy is to protect more effectively the marine environment across Europe. The MSFD requires that Member States take measures to achieve or maintain Good Environmental Status (GES) by 2020. The goal of the MSFD is in line with the objectives of the Water Framework Directive (WFD–2000/60/EC Directive [6]), achieving good ecological status by 2015, for surface water including marine-coastal waters and in case of chemical status, also territorial waters, and providing for the first review of the River Basin Management Plans in 2020 [7], [8]. MSFD Annex I includes a set of 11 descriptors, on the basis of which GES should be determined. Some descriptors deal with general status of the environment, others with specific habitat integrity or specific pressures. The Commission Decision of 1 September 2010 ([9] COM DEC 2010/477/EU) listed 29 criteria and 56 indicators, based on the scientific and technical assessment prepared by the Task Groups set up by the Joint Research Centre and the International Council on the Exploration of the Seas, to assess GES for each descriptor of the MSFD Annex I. These criteria and indicators include a factors’ combination in relation to state, impacts and pressures.

Member States had to make an initial assessment of own marine waters, taking account of existing data, where available, specifying the essential features and characteristics and current environmental status of the waters. The initial assessment was provided by 2012, together with the definition of targets and monitoring indicators. For this evaluation, it was necessary to point out predominant pressures and impacts existing in each region or subregion (MSFD art. 8, [4]). By 2015, Member States will have to develop coherent and coordinated programmes of measures. To ensure clean, healthy and productive marine waters, Member States strategies will have to be coordinated, consistent and properly integrated with regulations provided by existing Community legislation (such as transport, fisheries, tourism, infrastructure, research) and international agreements.

This work describes the initial assessment by means of criteria and indicators defined on Descriptor 9 (“*Contaminants in fish and other seafood for human consumption do not exceed levels established by Community legislation or other relevant standards*”), carried out by the Institute for Environmental Protection and Research (ISPRA) on behalf of the Italian Ministry of Environment.

### Focus on Descriptor 9: Contaminants in fish and other seafood for human consumption

MSFD Descriptor 9 requires to use health tools to assess the marine environment; it starts from ensuring that contaminants in fish and other seafood for human consumption, detected in the sea, do not exceed levels established by Community legislation or other relevant standards in order to protect the public health and, as indirect effect, to avoid negative influence on the sustainable use of marine resources. Thus, Good Environmental Status should be achieved when contaminants are below the levels fixed for human consumption, although the absence of human health effects may however involve environmental pollution effects. Descriptor 9 necessarily requires a balance between health information and environmental assessment; so, it becomes necessary to identify possible relations between contaminant levels in seafood tissues and the status of marine environment. In this way, starting from health information, it should be possible to reconstruct a general description of the environmental status of the area where the analyzed organisms live.

Decision 2010/477/EU [9] defines the need to monitor the presence of contaminants and hazardous substances in edible tissues of fisheries species fit for human consumption, by the use of two indicators:

9.1.1: actual levels of contaminants that have been detected and number of contaminants which have exceeded maximum regulatory levels;
9.1.2: frequency of regulatory levels being exceeded.

So, the goal of the Descriptor 9 is to check the public health. Nevertheless the aim of the Marine Strategy Directive is providing to the achievement of the Good Environmental Status, taking into account all descriptors together.

Indeed, also regarding data retrieval, we observed that existing national health monitoring programs often do not cover the information needs for compliance to GES, because monitoring programs on fish and seafood for public health reasons focus on estimating consumer exposure rather than assessing environmental status. Sampling procedures include all sizes of fish sold for human consumption rather than focusing on a standardized sample (offering greater possibilities in comparing degrees of contamination in the marine environment), without considering biological factors [10] that can influence concentrations of contaminants in fish, such as seasonal variation, age and sex [11]. However, the standardized sampling shuld produced a loss of representativeness compared to the great variability of samples available for human consumption.

Anyway, GES for Descriptor 9 should be judged in view of the monitoring of Descriptor 8 (“*Concentrations of contaminants are at levels not giving rise to pollution effects*”), also dealing with contaminants in marine environment. Descriptors 8 and 9 both measure contaminants in the marine environment, but focus on different state indicators. The importance of pay serious attention to the public health comes from the increasing recognition that the body burdens of environmental contaminants in the European human population is a growing cause for concern and may be associated with alterations in the frequency of occurrence of a range of diseases including diabetes, heart attacks, strokes, neurological disorders and cancers [12], [13]. Otherwise, the global per-capita demand for seafood has reached an all-time high, and is likely to continue to increase [14].

It was noted that almost all the contaminants thought to be of concern for marine vertebrates are also of concern for human health, due to the transfer along the food web. Thus, the relationship between the degree of chemical contamination and health of marine vertebrates is of great relevance to contaminant-related human diseases [15]. This is likely to be an even greater area of interest in the coming years as the human demographic ageing proceeds across Europe, allowing longer-term accumulation of contaminants in human tissues.

### Principles for assessing Italian marine waters

Concerning to Mediterranean area, there were some studies, most of them conducted in non-Italian waters, stale and with a very local profile (i.e. [16], [17], [18], [19], [20], [21]). Among them, European Environmental Agency published data (1985–2002) relating to concentrations of selected metals and organic contaminants in Mediterranean Sea mussels, highlighting a moderate variability and values non-exceeding regulatory levels. That being so, a more recent data collection was needed.

Italy used both results from health and environmental monitoring, so as to allow a proper evaluation and to cover the information needs for monitoring compliance to GES under Descriptor 9.

Human health programs monitored fish from different sizes and ages; so, higher levels can not automatically be interpreted as a negative status or evolution of the marine environment. In fact contamination appears to change according to the trophic level considered, with estimates higher in larger species, i.e. pelagic fish, containing more fat and occupying an higher food web position [22].

That being so, the “one out, all out” (OOAO) approach, based on the practice that the status of the worst element determines the final status, doesn’t seem applicable although it may be regarded as a precautionary approach [3], [7]. In case one species shows concentrations exceeding the regulatory levels, the (sub) region should be assessed as bad status for Descriptor 9; this judgment could easily be interpreted that consumption of all fish and seafood originating in that (sub) region would be dangerous.

Otherwise the Marine Strategy does not give specific rules, allowing Member States to adopt different criteria in order to use correctly health data for assessing environmental quality.

In Italy, a statistical range of acceptance was applied in order to avoid losing information, as reported below. Regarding the assessment of contaminants and the number of pollutants that exceeded law levels, Italy proposed that Good Environmental Status is achieved when concentrations are statistically lower than the thresholds identified by national and international legislation (Reg. 1881/2006 [23] and further updates [24], [25], [26], D.Lgs. 152/2006 [27]), estimated on samples of wild caught fish and aquaculture products (mussels) coming from national waters. Special attention was paid to assure traceability, selecting samples directly linked to the Italian (sub) regions laid down in the MSFD. The need arose since seafood is widely transported, its provenance may not be identifiable and human exposure monitoring programs often lack the necessary data to link the samples to specific (sub) regions. In addition, Italy, as Task Group 9 suggests, took care to make a selection of species, choosing those more prone to biomagnify/bio-accumulate regulatory classes of contaminants, species representative of the different trophic levels or habitats, species representative for different subregions.

## Methodology

Marine Strategy Directive divided European marine waters in 4 regions: Baltic, North East Atlantic Ocean, Mediterranean Sea and Black Sea. Some of them, as Mediterranean Sea, had been subdivided in subregions and Commission Decision of 1 September 2010 [9] established that Member States should performed initial assessment in each subregions of jurisdiction.

**Figure 1:**
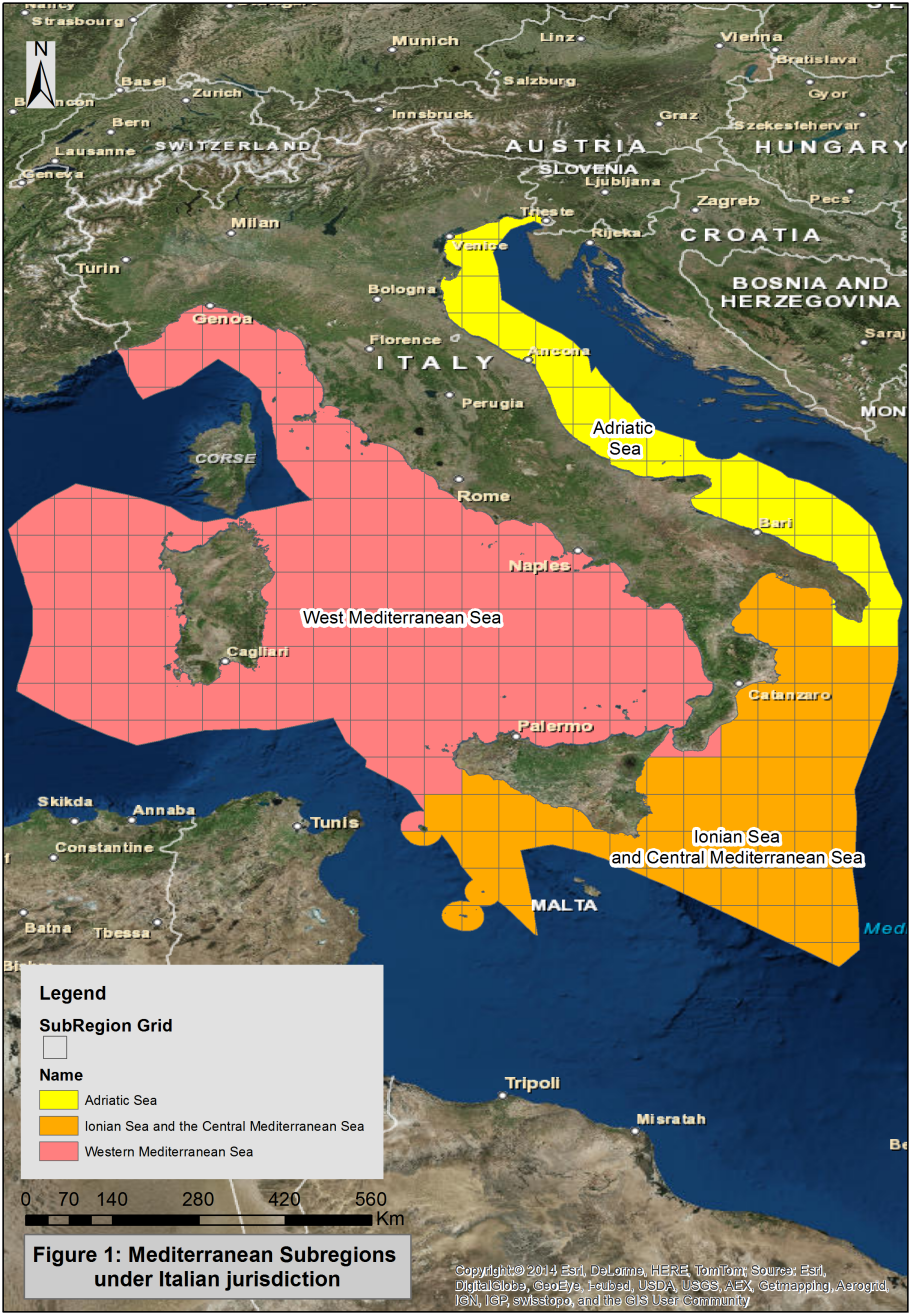
Mediterranean Subregions under Italian jurisdiction.

Italian waters fall into Mediterranean region and Western Mediterranean Sea (WMS), Adriatic Sea (AS) and Ionian Sea and the Central Mediterranean Sea (ISCMS) subregions are under Italian authority (Fig. 1).

The regulatory framework is essentially composed of Regulation (EC) No 1881/2006 [23] and further updates [24], [25], [26] on contaminants levels, set taking into account their toxicity as well as their prevalence in the food chain, and of Regulations (EC) No 882/2004 [28], No 1883/2006 [29] and No 333/2007 [30] (and respective further updates) regarding sampling procedures, methods of analysis and analytical quality. The Regulations provide for consumers protection from chemical substances in food and set maximum levels for certain contaminants in foodstuffs in order to keep contaminants at levels which are toxicologically acceptable. Each regulated contaminant has a different threshold level, laid down according to the specific tissue analyzed. Descriptor 9 refers to these regulations, but also to other relevant national standards (in Italy i.e. D.Lgs. 152/2006 fixes mercury and lead levels in mussel tissues, concerning to water quality required for mussel farms).

Chemical contaminants monitored were those for which regulatory levels have been laid down for public health reasons: heavy metals (lead, cadmium, and mercury), polycyclic aromatic hydrocarbons (benzo(a)pyrene, benz(a)anthracene, benzo(b)fluoranthene and chrysene) and dioxins (including dioxin-like PCBs). Each contaminant was identified by category code reported in Reg. 1881/2006 [23], Reg. 835/2011[24] and Reg. 1259/2011 only for dioxins and dioxin-like PCBs [26], providing for specific values of edibility, relative to different species and analyzed tissues. In table 1, regulatory values, reference legislation, code and foodstuff categories are summarized.

**Table 1:** Regulatory levels, reference legislation, code and foodstuff categories.

Category code	Legislation	Foodstuff	Regulatory levels
**Cd 3.2.5**	Reg.1881/2006/CE	Muscle meat of fish (footnote 24)	0,05 mg/kg w.w.
**Cd 3.2.6**	Reg.1881/2006/CE	Muscle meat of listened fish	0,10 mg/kg w.w.
**Cd 3.2.8**	Reg.1881/2006/CE	Crustaceans	0,50 mg/kg w.w.
**Cd 3.2.9**	Reg.1881/2006/CE	Bivalve molluscs	1,0 mg/kg w.w.
**Cd 3.2.10**	Reg.1881/2006/CE	Cephalopods	1,0 mg/kg w.w.
**Hg 3.3.1**	Reg.1881/2006/CE	Fishery products and musclemeat of fish (footnotes 24, 25, 26)	0,50 mg/kg w.w.
**Hg 3.3.2**	Reg.1881/2006/CE	Muscle meat of listened fish	1,0 mg/kg w.w.
**Pb 3.1.5**	Reg.1881/2006/CE	Muscle meat of fish (footnote 24)	0,3 mg/kg w.w.
**Pb 3.1.6**	Reg.1881/2006/CE	Crustaceans	0,50 mg/kg w.w.
**Pb 3.1.7**	Reg.1881/2006/CE	Bivalve molluscs	1,5 mg/kg w.w.
**Pb 3.1.8**	Reg.1881/2006/CE	Cephalopods	1,0 mg/kg w.w.
**Dioxins 5.3**	Reg.1259/2011/CE	Muscle meat of fish and Bivalve molluscs	3,5 pg/g w.w.
**Sum dioxins and** **dioxin like PCBs 5.3**	Reg.1259/2011/CE	Muscle meat fish and Bivalve molluscs	6,5 pg/g w.w.
**Benzo(a)pyrene 6.1.4**	Reg.1881/2006/CE	Muscle meat of fish (footnote 24)	2,0 µg/kg w.w.
**Benzo(a)pyrene 6.1.5**	Reg.1881/2006/CE	Crustaceans and Cephalopods	5,0 µg/kg w.w.
**Benzo(a)pyrene 6.1.6**	Reg.835/2011/CE	Bivalve molluscs	5 µg/kg w.w.
**Sum PAH 6.1.6**	Reg.835/2011/CE	Bivalve molluscs	30 µg/kg w.w.

It was noted that almost all the contaminants thought to be of concern for marine vertebrates are also of concern for human health, due to the transfer along the food web. Thus, the relationship between the degree of chemical contamination and health of marine vertebrates is of great relevance to contaminant-related human diseases [15]. This is likely to be an even greater area of interest in the coming years as the human demographic ageing proceeds across Europe, allowing longer-term accumulation of contaminants in human tissues.

For each contaminant, the number of excesses and their frequency on the total number of observed data were indicated.

### Calculation of statistical range of acceptance

Based on the thresholds identified in the relevant national and international laws, statistical ranges of acceptance were calculated for the concentrations of regulated contaminants.

To calculate these ranges, it was utilized R software. The assumption used is that contaminants’ concentrations follow lognormal distribution Y∼LN(µ, σ) [31], [32], with parameter µ equal to the geometric mean of collected contaminant’s data and σ equal to 20% of this value, as required for environmental data [33]. We have also tested this assumption, with Shapiro Normality test on logarithm of actually used data. In order to identify statistical ranges, it was assumed that threshold level (TL) is the 90th percentile of theoretical distribution and that 90th percentile of empirical data distribution must not exceed this level.

### GIS elaborations

Useful data for the assessment were georeferenced in GIS (Software ArcGis 10.1) and exported into Feature Classes, taking as reference system ETRS89. The Feature Classes have been created on the basis of regulatory codes. The data were processed both for a single year, in order to see the concentration trend in the subregion, and considering the whole period (2006–2012), as requested by MSFD. Polygonal grids were created to cover the entire subregion.

A critical aspect of the cartographic layers production was the definition of the reference grid (fishnet) by selecting:

- The starting point (lower left corner);
- the cell size(s);
- the number of rows and columns.

As in 2009 ISPRA was involved in the EuSeaMap project (Mapping European seabed habitats, [34]), in which a broad-scale modelled habitat map for the Western Mediterranean was produced together with the Spanish and French partners, the fishnet was created according to the grid used in this project.

GIS layers were created in geographic coordinates (ETRS 89), using angular measures in order to obtain a 1∶1 cell size ratio. The chosen mesh was 50 km per side, coherently with the ecological differences (such as mobility, migratory patterns and feeding habits) of functional groups and habitats considered.

Value of “code” 1 and green colour’s cell were assigned to data not exceeding the threshold levels. Value of “code” 2 and red colour’s cell were assigned to data above reference thresholds.

The stations results which fall in the same cell and/or which have the same coordinates (e.g. data relating to the same station, but sampled in different years or related to differences species belonging to the same regulatory category) were integrated, as follows:

if stations number with code 1 and 2 is different, the cell has code with the highest frequency.
if stations number with code 1 is equal to stations number with code 2, the cell reaches code 2, in compliance with the precautionary principle [7];

So, in the cartographic representation, colour green had been assigned to cells with majority of station with code 1, while colour red to the cells with equal or majority of station with code 2.

For the three subregions, the percentage of spatial coverage for individual contaminants and the percentage of stations that exceed the limit (frequency of exceedances) were determined.

No review from the ethic committee was required as our research work did not involve any direct manipulation or disturbance of animals. Blood samples were collected by and under the permit of the Israel Nature and Parks Authority. Access to all sampling locations was not restricted and so no specific permissions were required.

## Results and Discussion

### Initial Assessment

The initial assessment examined substances for which maximum levels are established in fish, shellfish and other marine products destined for human consumption, defined a statistical range of acceptance, calculated the number and frequency of contaminants exceeding maximum regulatory levels and handed over these information to environmental quality assessment.

In the Mediterranean Sea approximately 15000 data were collected, concerning Metals (Hg, Cd, Pb), PAHs (benzo(a)pyrene and sum of benzo(a)pyrene, benz(a)anthracene, benzo(b)fluoranthene and chrysene) and dioxins (including dioxin-like PCBs).

Data related to Adriatic Sea were 7119 of which 5801 for Metals, 1086 for PAHs and 232 for HOCs.

In West Mediterranean Sea 1900 data were examined, of which 1344 for Metals, 513 for PAHs and 43 for HOCs. For Ionian Sea and Central Mediterranean Sea the number of analyzed data was equal to 5983, of which 5865 for Metals, 92 for PAHs and 26 for HOCs.

Data were elaborated and those unfitting in analytical quality (Annex III of Commission Regulation (EC) No 882/2004 [28]), in MSFD criteria and in GIS elaborations standards, were removed. Collected data concerned edible portion of different target species both from environmental and commercial value point of view, but also representing a coverage of the entire subregion as better as possible. Target species were principally coastal fish, tuna, mullet, mussels, shark, crustaceans, cephalopods, selected using Annex II of Task Group 9 Report [35]. For PAHs’, bivalve molluscs were used as preferred species for initial assessment, being sedentary benthic species with a large lipid content [36], [37].

Tables 2–4 show statistical outputs for each Subregion. Percentage of spatial coverage and percentage of data, stations and cells, within or outside regulatory limits, are reported. Regulatory levels (Tab. 1) are referred to categories established by Reg. 1881/2006 [23]. Limits of the sum of PAHs and benzo(a)pyrene in bivalve molluscs related to Reg. 835/2011/CE [24] while limits for s and dioxin like PCBs for muscle meat of fish and bivalve molluscs referred to Reg. 1259/2011/CE [26].

**Table 2:** Statistical outputs for Metals.

AS* (total cells 51)	Cd 3.2.5	Cd 3.2.6	Cd 3.2.8	Cd 3.2.9	Cd 3.2.10	Hg 3.3.1	Hg 3.3.2	Pb 3.1.5	Pb 3.1.7	Pb 3.1.8	Pb 3.1.6
**% of spatial coverage**	**27.5**	**11.8**	**5.9**	**47.1**	**9.8**	**82.4**	**11.8**	**33.3**	**49.0**	**9.8**	**5.9**
**% of data within the limits**	100	100	100	99.4	100.0	99.4	93.8	92.9	99.7	100	100
**% of data outside the limits**				0.6		0.6	6.2	7.1	0.3		
**% of station within the limits**	100	100	100	100	100	97.3	85.7	94.7	100	100	100
**% of station outside the limits**						2.7	14.3	5.3			
**% of cells within the limits**	100	100	100	100	100	95.2	83.3	94.1	100	100	100
**% of cells outside the limits**						4.8	16.7	5.9			
**WMS** ** **(total cells** **166)**	**Cd 3.2.5**	**Cd 3.2.6**	**Cd 3.2.8**	**Cd 3.2.9**	**Cd 3.2.10**	**Hg 3.3.1**	**Hg 3.3.2**	**Pb 3.1.5**	**Pb 3.1.7**	**Pb 3.1.8**	**Pb 3.1.6**
**% of spatial coverage**	**3.6**	**2.4**	**-**	**10.8**	**-**	**15.1**	**3.6**	**7.8**	**11.4**	**-**	**-**
**% of data within the limits**	85	75	-	100	-	88.3	87.5	87.2	96	-	-
**% of data outside the limits**	15	25	-		-	11.7	12.5	12.8	4	-	-
**% of station within the limits**	85.7	75	-	100	-	92.3	85.7	86.7	97.7	-	-
**% of station outside the limits**	14.3	25	-		-	7.7	14.3	13.3	2.3	-	-
**% of cells within the limits**	83.3	75	-	100	-	96	83.3	92.3	94.7	-	-
**% of cells outside the limits**	16.7	25	-		-	4	16.7	7.7	5.3	-	-
**ISCMS** *** **(total cells** **77)**	**Cd 3.2.5**	**Cd 3.2.6**	**Cd 3.2.8**	**Cd 3.2.9**	**Cd 3.2.10**	**Hg 3.3.1**	**Hg 3.3.2**	**Pb 3.1.5**	**Pb 3.1.7**	**Pb 3.1.8**	**Pb 3.1.6**
**% of spatial coverage**	**5.2**	**5.2**	**-**	**5.2**	**-**	**7.8**	**9.1**	**7.8**	**3.9**	**-**	**-**
**% of data within the limits**	100	75	-	100	-	100	100	100	99.8	-	-
**% of data outside the limits**		25	-		-				0.2	-	-
**% of station within the limits**	100	60	-	100	-	100	100	100	100	-	-
**% of station outside the limits**		40	-		-					-	-
**% of cells** **within the limits**	100	50	-	100	-	100	100	100	100	-	-
**% of cells outside the limits**		50	-		-					-	-

* Adriatic Sea SubRegion;

** West Mediterranean Sea SubRegion;

*** Ionian Sea and Central Mediterranean Sea SubRegion.

**Table 3:** Statistical outputs for PAHs (Polycyclic Aromatic Hydrocarbons).

AS* (total cells 51)	Benzo(a)pyrene6.1.4	Benzo(a)pyrene6.1.5	Benzo(a)pyrene6.1.6	Sum of PAHs6.1.6
**% of spatial coverage**	**2**	**-**	**29.4**	**29.4**
**% of data within the limits**	100	-	100	100
**% of data outside the limits**		-		
**% of station within the limits**	100	-	100	100
**% of station outside the limits**		-		
**% of cells within the limits**	100	-	100	100
**% of cells outside the limits**		-		
**WMS** ** **(total cells** **166)**	**Benzo(a)pyrene** **6.1.4**	**Benzo(a)pyrene** **6.1.5**	**Benzo(a)pyrene** **6.1.6**	**Sum of** **PAHs 6.1.6**
**% of spatial coverage**	**3.6**	**1.2**	**3.6**	**4.8**
**% of data within the limits**	100	100	94.3	98.7
**% of data outside the limits**			5.7	1.3
**% of station within the limits**	100	100	89.7	96.3
**% of station outside the limits**			10.3	
**% of cells within the limits**	100	100	66.7	88.9
**% of cells outside the limits**			33.3	
**ISCMS** *** **(total cells** **77)**	**Benzo(a)pyrene** **6.1.4**	**Benzo(a)pyrene** **6.1.5**	**Benzo(a)pyrene** **6.1.6**	**Sum of PAHs** **6.1.6**
**% of spatial coverage**	-	-	**2.6**	**2.6**
**% of data within the limits**	-	-	85.7	94.4
**% of data outside the limits**	-	-	14.3	5.6
**% of station within the limits**	-	-	100	100
**% of station outside the limits**	-	-		
**% of cells within the limits**	-	-	100	100
**% of cells outside the limits**	-	-		

* Adriatic Sea SubRegion;

** West Mediterranean Sea SubRegion;

*** Ionian Sea and Central Mediterranean Sea SubRegion.

**Table 4:** Statistical outputs for Dioxins and Dioxins like PCBs.

AS*( total cells 51)	Dioxins 5.3	Sum Dioxins and dioxin like 5.3
**% of spatial coverage**	-	**56.9**
**% of data within the limits**	-	100
**% of data outside the limits**	-	
**% of station within the limits**	-	100
**% of station outside the limits**	-	
**% of cells within the limits**	-	100
**% of cells outside the limits**	-	
**WMS** ** **(total cells** **166)**	**Dioxins 5.3**	**Sum Dioxins and dioxin like 5.3**
**% of spatial coverage**	**1.2**	**4.2**
**% of data within the limits**	100	92.9
**% of data outside the limits**		7.1
**% of station within the limits**	100	100
**% of station outside the limits**		
**% of cells within the limits**	100	100
**% of cells outside the limits**		
**ISCMS** *** **(total cells** **77)**	**Dioxins 5.3**	**Sum Dioxins and dioxin like 5.3**
**% of spatial coverage**	**1.3**	**1.3**
**% of data within the limits**	87.5	100
**% of data outside the limits**	12.5	
**% of station within the limits**	85.7	100
**% of station outside the limits**	14.3	
**% of cells within the limits**	100	100
**% of cells outside the limits**		

* Adriatic Sea SubRegion;

** West Mediterranean Sea SubRegion;

*** Ionian Sea and Central Mediterranean Sea SubRegion.

Italy tried to hand over health information in terms of environmental assessment, without judging the outcome of a single sample, as requested by human consumption legislations, but integrating the results of all stations included into the same cell. For each regulatory category, different integration levels were performed: data within the same station; several stations falling in the same grid cell; same grid cell in different years.

The analyzed data showed that inorganic elements (metals) are usually more investigated than organic contaminants; indeed for metals the spatial coverage reaches 82% in AS subregion, while the maximum PAHs and sum Dioxins-dioxin like PCBs percentages are 29 and 57 respectively, still in AS subregion.

These data showed that the favourite organisms to assess the health risk are generally bivalve molluscs, as demonstrated by spatial coverage percentage of Cd 3.2.9, Hg 3.3.1(chiefly populated by molluscs), Pb 3.1.7 and BaP 6.1.6. Indeed, the mussel *Mytilus galloprovincialis* has all the desirable characteristics of a biological indicator and is the main species recommended to perform bio-monitoring activities; moreover, both the biology and ecology of the species are documented [38]. For these reasons mussel watch programs are widely diffused to evaluate environmental quality of marine water [38], [39], [40]. On the other hand, sedentary benthic species, having a high lipid content, are suggested as preferred species for PAHs’ monitoring activities on human consumption [37], [41].

Adriatic Sea (AS) subregion data referred to bivalve mollusc organisms, fishes (coastal fish, tuna, mullet, shark), crustaceans and cephalopods. In the Ionian Sea and Central Mediterranean Sea (ISCMS) subregion data distribution included mullet, nectobentonic fishes, bivalve molluscs, anchovies, sardines and seabream. Concerning Western Mediterranean (WMS) subregion, bivalve molluscs, crustaceans, mullet, seabream, seabass and flatfishes were studied.

**Figure 2:**
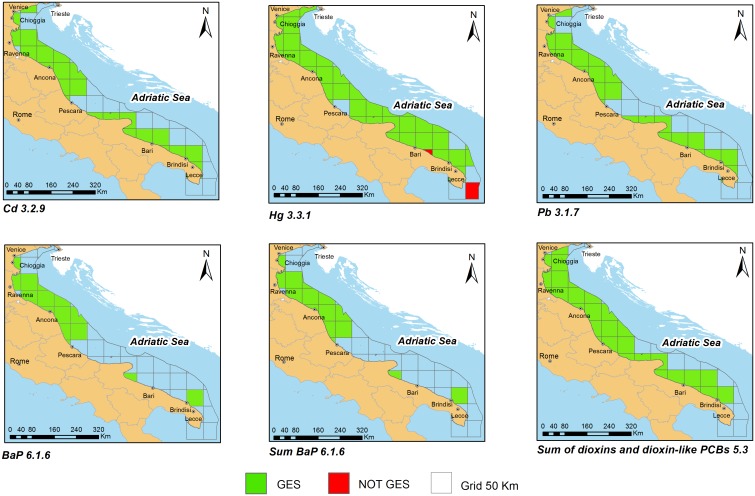
Results on Metals, PAH and Dioxins/Dioxin-like PCBs in Adriatic Sea Subregion (AS).

**Figure 3:**
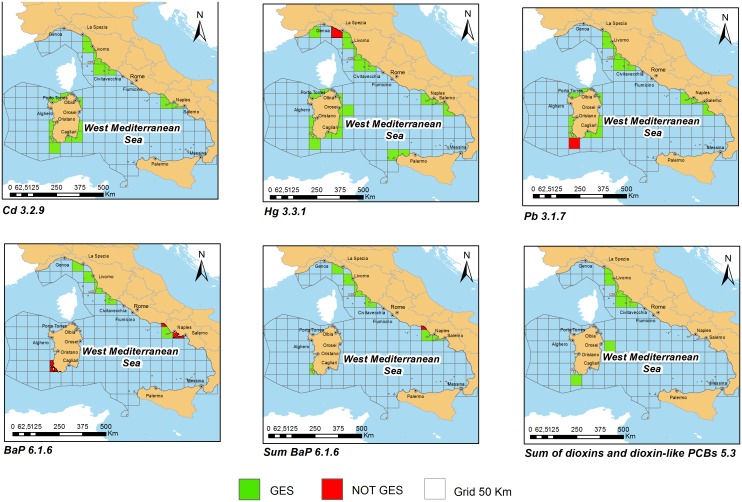
Results on Metals, PAH and Dioxins/Dioxin-like PCBs in Western Mediterranean (WMS).

Results on Metals, PAH and Dioxins/Dioxin-like compounds concerning to the seafood categories with greater spatial coverage are reported in figures 2 and 3. ISCMS results are not shown as the spatial coverage of data was very low compared to the number of subregion total cells.

Data were collected especially in coastal zones, where the main fishing activities take place; so the spatial coverage was often lacking in the open sea.

After integration, in all subregions, the environmental quality by metals was good, although some stations exceeded regulatory levels. In the AS subregion, Pb 3.1.5, and Hg 3.3.2 exceeded in muscle of twaite shad (*Alosa fallax*) and albacore tuna (*Thunnus alalunga*) while Hg 3.3.1 exceeded in blackbelly rosefish (*Helicolenus dactylopterus*), in thornback ray (*Raja clavata*) and in shellfish tissues. In the WMS subregion several stations exceeded regulatory levels concerning all codified categories of metals, except Cd 3.2.9. Indeed Pb 3.1.5–3.1.7 passed limits in fish muscle of seabream (*Dicentrarchus labrax*) and shells, Hg 3.3.1 in bivalves and Hg 3.3.2 in Atlantic bluefin tuna (*Thunnus thynnus)* and finally Cd 3.2.5–3.2.6 in fish muscle of seabream and in European anchovy (*Engraulis encrasicolus*). In ISCM subregion all data were below regulatory levels except Cd 3.2.6 in European anchovy, that covered only about 5% of the whole subregion.

These exceedances confirm the literature data regarding the different metal behaviour due to propensity to undergo biomagnification and to trophic level and foraging method/location of organisms. Thus, it is not surprising that tuna, that is at high trophic levels, had some excesses of mercury and that bottom feeding fishes had the excesses too. Animals living in close association with sediments, in which they bury and from where they mainly feed, are, in fact, more exposed to any sediment-associated contamination than other fish [42], [43], [44]. Another relevant point when evaluating the variability in data is the fish size. There is, in fact, a large body of data showing that mercury tissue concentrations increase with age/size of marine fauna [45], [46], [47]. Concerning Cd and Pb, the concentration ranges were quite narrow and similar, with exception of data from industrial sites in the coastal areas of ISCM and WMS subregions, suggesting that there is no trend in tissue residues of these metals based on taxonomic position or trophic status [48].

However, independently from the interaction complexity leading to a different toxic load among species, collected metals concentrations were in agreement with those reported in seafood from either the same environment [43], [44], [49] or other marine areas [50].

Concerning dioxins and dioxin-like compounds, in the AS and WMS subregions data were below the regulatory thresholds, while in the ISCMS only data referred to mussels (*Mytilus galloprovincialis*) showed some excesses; anyway the grid cells were all within the limits. The spatial cover for dioxins and dioxin-like compounds was higher in Adriatic subregion (56,9%), where the most part of mussel farming sites are located [51], while in the others subregions the coverage didn’t reach 5% overall.

The monitoring of benzo(a)pyrene and the others congeners (benz(a)anthracene, benzo(b)fluoranthene and chrysene) was carried out in a systematic way in Adriatic Sea subregion, where data collected covered about 30% of the subregion and the environmental quality was good. Despite of that, in Western Mediterranean Sea and Ionian Sea and Central Mediterranean subregions the coverage percentage didn’t go beyond 5% and some data in bivalve molluscs exceeded the regulatory thresholds for benzo(a)pyrene. It is well known that seasonal differences in biotic and abiotic factors regulate metabolic mechanisms and PAHs bioaccumulation and that bivalve molluscs are not able to metabolize PAHs, accumulating them in their organisms [49]. Nevertheless, several works described annual cyclic variation in the PAHs content of seafood, so that data from *Mytilus galloprovincialis* collected in winter exceeded regulatory levels [41], [52], [53].

Despite some exceedances, environmental quality of Italian subregions was good overall, regarding Metals, PAHs, Dioxins and Dioxin like compounds, taking into account coverage percentages too. Indeed, concentrations over thresholds were linked not only to the presence of the pollutant, but also to a whole range of biological (species, age, growth degree) and environmental (temperature, geochemical anomalies, salinity) factors which influence trace elements bioavailability [49], [54], [55], [56]. Human health programmes effectively do not contemplate seasonal variation, age and sex which influence contaminants’ bioavailability in fish and seafood. So higher levels of contaminants concentrations should not automatically be interpreted as a negative status.

## Conclusion

The analysis of the collected data allowed to carry out the initial assessment on environmental quality of the 3 Italian subregions of the Mediterranean Sea. Results showed that the three subregions were overall in good status, since, for each substance or category, the greater part of the investigated cells had a concentration statistically lower than the acceptance range calculated assuming the regulatory levels as the 90 percentile of data distribution. However, it is needed to underline that data spatial coverage is limited, especially for synthetic compounds and, for this reason, results could have some limitations.

For this initial assessment Italy used data from health and environmental monitoring plans because existing national health monitoring programs didn’t not cover all information needs for monitoring compliance to GES under Descriptor 9. It would be desirable improve monitoring activities considering Descriptor 9 in conjunction with requirements for Descriptor 8. Moreover it would be interesting to identify possible relations between contaminant levels in sediment and biota’s, in order to explain the transfer of contaminants from sediment to biota and in order to establish a well-defined quantitative link between levels in fish and other seafood and levels of contaminants in marine environment. That might help to clearly demonstrate the transfer of them from marine environment to fish/fishery species and, at the end, to man.

So that researchers could best benefit from future monitoring programs (art.11 MSFD) and from information obtained, major adaptations would be needed regarding sampling plans’ design, sampling procedures, matrix on which to perform the analysis as well as traceability to the location of catching or harvesting. In fact monitoring programs on human exposure use regulatory levels set for public health, but they often lack the necessary information to link the samples and results to specific subregions. In addition, these monitoring programs check out contaminants for which regulatory levels have been set, while other contaminants of relevance in fish and other seafood, distinguishing of a specific area, activity or input source, should be investigated to evaluate both spatial and temporal trends [35]. It would be opportune to set a limit also for total PAHs, organochlorine pesticides and organotin compounds. Infact, to date, the legislative Regulation 835/2011/CE [24] provides only a limit for the sum of 4 PAHs. Among toxic metals, only Pb, Cd and Hg have a limit set by E.C. Reg. 1881/2006 [23], while other metals also might become toxic when elevated doses are ingested [37], [57].

Nevertheless there is still the need to improve the flow of information coming from institutions working in the health field, in order to validate the applied methodology and confirm the possibility to extend the use of health information arising from chemical contamination of seafood for the evaluation of the quality of the marine environment.

This shrewdness will be functional to fill part of the knowledge gaps that emerged from the initial assessment and nevertheless indispensable for a better definition of GES and Targets.
